# Racial variation in lipoprotein-associated phospholipase A_2 _in older adults

**DOI:** 10.1186/1471-2261-11-38

**Published:** 2011-06-29

**Authors:** Keane K Lee, Stephen P Fortmann, Ann Varady, Joan M Fair, Alan S Go, Thomas Quertermous, Mark A Hlatky, Carlos Iribarren

**Affiliations:** 1 Department of Health Research and Policy, Stanford University School of Medicine, HRP Redwood Building, Stanford, CA 94305-5405 USA; 2 Kaiser Santa Clara Cardiology, Department 348, Kaiser Permanente of Northern California, 710 Lawrence Expressway, Santa Clara, CA 95051 USA; 3 Center for Health Research, Kaiser Permanente Northwest, 3800 N. Interstate Avenue, Portland, OR 97227 USA; 4 Stanford Prevention Research Center, Stanford University School of Medicine, Medical School Office Building, 251 Campus Drive, Stanford, CA 94305-5411 USA; 5 Division of Research, Kaiser Permanente of Northern California, 2000 Broadway, Oakland, CA 94612 USA; 6 Department of Epidemiology, Biostatistics, and Medicine, University of California, San Francisco, 185 Berry Street, Lobby 5, Suite 5700, San Francisco, CA 94107 USA; 7 Cardiovascular Medicine, Stanford University School of Medicine, 300 Pasteur Drive, Falk Cardiovascular Research Center, Stanford, CA 94305-5406 USA

## Abstract

**Background:**

Lipoprotein-associated phospholipase A_2 _(Lp-PLA_2_) is a predictor of cardiovascular events that has been shown to vary with race. The objective of this study was to examine factors associated with this racial variation.

**Methods:**

We measured Lp-PLA_2 _mass and activity in 714 healthy older adults with no clinical coronary heart disease and not taking dyslipidemia medication. We evaluated the association between race and Lp-PLA_2 _mass and activity levels after adjustment for various covariates using multivariable linear regression. These covariates included age, sex, diabetes, hypertension, body mass index, lipid measurements, C-reactive protein, smoking status, physical activity, diet, income, and education level. We further examined genetic covariates that included three single nucleotide polymorphisms shown to be associated with Lp-PLA_2 _activity levels.

**Results:**

The mean age was 66 years. Whites had the highest Lp-PLA_2 _mass and activity levels, followed by Hispanics and Asians, and then African-Americans; in age and sex adjusted analyses, these differences were significant for each non-White race as compared to Whites (p < 0.0001). For example, African-Americans were predicted to have a 55.0 ng/ml lower Lp-PLA_2 _mass and 24.7 nmol/ml-min lower activity, compared with Whites, independent of age and sex (p < 0.0001). After adjustment for all covariates, race remained significantly correlated with Lp-PLA_2 _mass and activity levels (p < 0.001) with African-Americans having 44.8 ng/ml lower Lp-PLA_2 _mass and 17.3 nmol/ml-min lower activity compared with Whites (p < 0.0001).

**Conclusion:**

Biological, lifestyle, demographic, and select genetic factors do not appear to explain variations in Lp-PLA_2 _mass and activity levels between Whites and non-Whites, suggesting that Lp-PLA_2 _mass and activity levels may need to be interpreted differently for various races.

## Background

Lipoprotein-associated phospholipase A_2 _(Lp-PLA_2_) circulates in the blood as an enzyme bound mainly to low-density lipoprotein (LDL) particles. Lp-PLA_2 _has been found in multiple studies to be associated with incident and prevalent coronary heart disease (CHD)[1-6] and incident stroke,[5,7] independent of standard cardiovascular risk factors[8]. A collaborative meta-analysis of 32 prospective studies in 79,000 patients showed that both Lp-PLA_2 _mass and activity added predictive value for vascular events and mortality beyond traditional cardiovascular risk factors[9]. Currently, a direct Lp-PLA_2 _inhibitor is being tested in a randomized trial for prevention of cardiovascular events in patients with CHD[10]. In a study of the general population, Lp-PLA_2 _mass and activity levels were higher in Whites compared with African-Americans or Hispanics[11]. However, the degree to which genetic differences as opposed to lifestyle variations, including smoking status, diet and exercise, may explain racial differences remains unclear. The goals of this study were to confirm the racial variations in Lp-PLA2 mass and activity levels and to examine the factors that might explain these racial differences in a multi-racial cohort of healthy older individuals free of clinical CHD and not taking dyslipidemia medications.

## Methods

### Study Sample

The participants in this study were recruited from Kaiser Permanente Northern California, a large integrated healthcare system that provides comprehensive care to more than 3 million people in San Francisco and the greater Bay Area. Details of participant recruitment have been described previously[12,13]. Briefly, we enrolled adults between 60 and 72 years old who lived within 50 miles of the research facility, and who did not have a diagnosis of cardiovascular disease, cancer, end-stage renal disease, liver failure, dementia, or human immunodeficiency virus infection. By January 2001, 3,054 apparently eligible subjects were randomly chosen to be sent letters inviting participation. From these invitations, a total of 1,024 subjects were recruited, consisting of 639 men and 385 women. In the final year of recruitment, men, Hispanics, and African-Americans were selectively oversampled in order to better match the cases enrolled in the Atherosclerotic Disease, Vascular Function, and Genetic Epidemiology study[14]. After excluding people who were taking dyslipidemia medications from this analysis, 714 subjects were included in the study. The study protocol was reviewed and approved annually by the institutional review boards at both Stanford University and the Division of Research at Kaiser Permanente Northern California. Written informed consent was obtained from all participants.

### Risk Factor Measurement

All the study subjects attended a research clinic visit at Stanford University and completed a health survey about medical diagnoses, medication use, smoking history, family history, race and ethnicity, dietary intake, and physical activity. Subjects were asked to bring their medications, which were independently reviewed and recorded by the interviewer. Resting blood pressure was determined using standard sphygmomanometers. Weight, height, and waist circumference were measured by trained and certified staff using a stadiometer, balance scale, and tape. Blood tests were drawn for biomarker analysis, which included fasting lipid levels, glucose, C-reactive protein,[15] and Lp-PLA_2 _mass and activity levels[16,17].

### Stanford Seven-Day Physical Activity Recall

The Stanford Seven-Day Physical Activity Recall, a semi-structured interview, was used to estimate the amount of time that a person engaged in moderate-, hard-, and very hard-intensity activities during the previous seven days[18,19]. A trained interviewer guided the subject through the recall process, day-by-day, to determine the duration and intensity of physical activities performed, as well as time spent sleeping[19]. Time spent in light activity was estimated by subtracting the time included in sleep and moderate-, hard-, and very hard-intensity activities from the total hours in the recall period. Total energy expenditure was estimated by multiplying the hours spent in each level of activity with the estimated energy expenditure value for each intensity category.

### Block Food Frequency Questionnaire

The Block Food Frequency Questionnaire was used to characterize dietary intake[20]. It queries average consumption of 106 foods by portion size and includes 13 questions on dietary supplementation, six questions on restaurant eating, five summary questions, eight questions on fat use or low-fat foods, and seven demographic and health-related questions.

### Race Determination

Self-reported race was collected during the eligibility screening survey, on the baseline health survey and from the Kaiser Permanente data sources. In addition, birthplace, grandparents' race, and grandparents' country of origin were collected. The algorithm for assigning race was as follows: if self-reported race and grandparents' race were concordant, then subjects were coded to that race category (> 80% of subjects). In discordant cases, race was assigned by a hierarchy of grandparents' race, grandparents' country of origin, self reported race on the baseline health survey, and self-reported race on the screening survey. Only the major racial groups of White, African-American, Hispanic and Asian are used for this analysis. This method of classifying race has been validated in this cohort by high-throughput genotyping of more than 450,000 single nucleotide polymorphisms (SNPs) among African-Americans and Whites[21].

### Lp-PLA_2 _Measurement

Blood samples were collected at the baseline visit after an overnight fast and stored in aliquots frozen at -80 °C. Lp-PLA_2 _mass (ng/ml) was measured using a dual enzyme linked immunoassay (PLAC test, diaDexus Inc., South San Francisco, CA)[16]. Intra- and inter-assay coefficients of variation were < 5% and < 8%, respectively, and sensitivity across the assay range was < 0.5 ng/mL. Lp-PLA_2 _activity (nmol/ml-min) was measured by a colorimetric activity method (CAM test)[17]. Intra- and inter-assay coefficients of variation were < 4% and < 6%, respectively, and sensitivity across the assay range was < 5 nmol/ml-min.

### Single Nucleotide Polymorphism Selection

A literature search for all of the documented SNPs related to Lp-PLA_2 _mass and activity levels was conducted. Only those SNPs that were both documented in the literature to be related to Lp-PLA_2 _and existed in our database were included in the analysis. The SNPs included in our database were selected for likely association with atherosclerosis as determined *a priori *from literature review and by comparing up and down-regulated genes in diseased and non-diseased vascular endothelium. The genetic covariates included three SNPs in the PLA2G7 locus (Lp-PLA_2 _gene) that have been documented to be associated with Lp-PLA_2 _activity: Ala379Val (rs1051931), Arg92His (rs1805017), and Ile198Thr (rs1805017)[22]. The SNPs were coded as 0 for homozygous major, 1 for heterozygous, and 2 for homozygous minor.

### Statistical methods

Baseline characteristics were presented as means and standard deviations for normally distributed continuous variables and as counts and proportions for categorical variables. Medians and interquartile ranges were used for non-normally distributed continuous variables. Differences in the baseline variables between racial groups were compared using a general linear model analysis of variance (ANOVA).

Linear regression was used to examine the association between race and Lp-PLA_2 _mass and activity in separate models. The covariates for multivariable analysis were selected *a priori *and included biological, lifestyle, demographic, and genetic characteristics. The biological covariates included age, race, history of diabetes mellitus, history of hypertension, quantitative systolic blood pressure measurement, body mass index (BMI), waist circumference, LDL cholesterol level, high density lipoprotein (HDL) cholesterol level, asymmetric dimethylarginine (ADMA), and C-reactive protein (CRP); the lifestyle and demographic covariates were current smoking status, physical activity, percent of calories from saturated fat, percent of calories from carbohydrates, education level, and income.

A simple linear regression model was first used to analyze the relation between race and Lp-PLA_2 _mass and activity adjusted for only sex. The biological, lifestyle, and genetic covariates were each included in separate multivariable models in addition to a single multivariable model with all the covariates. Data were analyzed using SAS, version 9.1 (SAS Institute, Cary, North Carolina).

## Results

The study population consisted of 540 whites, 60 African-Americans, 62 Hispanics, and 52 Asians (Table 1). The average age was 66 years and women comprised 40% of the study cohort. The prevalence of hypertension (62%) was slightly higher and the prevalence of diabetes (13%) relatively lower than the general population[23,24]. Mean LDL levels were mildly elevated (128 mg/dL), while mean HDL levels were relatively high (54 mg/dL). The cohort was overweight (mean BMI of 28 kg/m^2^) and only a small proportion were active smokers (7.3%).

**Table 1:** Baseline characteristics among older adults without clinical cardiovascular disease by major race subgroups; mean (s.d.) and N (%)

Variable	All subjects	White	African- American	Hispanic	Asian	P-value*
	(n = 714)	(n = 540)	(n = 60)	(n = 62)	(n = 52)	
Age (years)	65.8 (2.9)	65.7 (2.9)	65.5 (3.0)	66.3 (2.8)	65.9 (2.5)	0.38
Female gender	284 (40%)	216 (40%)	20 (33%)	26 (42%)	22 (42%)	0.73
Diabetes mellitus	91 (13%)	58 (11%)	15 (25%)	10 (16%)	8 (15%)	0.011
History of hypertension	440 (62%)	321 (59%)	45 (75%)	39 (63%)	35 (67%)	0.095
Systolic blood pressure (mmHg)	131 (18)	130 (17)	136 (22)	133 (15)	136 (20)	0.0045
Body mass index (kg/m^2^)	27.9 (5.0)	27.8 (5.0)	30.3 (5.8)	28.9 (3.9)	25.4 (3.6)	< 0.0001
Waist circumference (cm)	93.0 (14.6)	92.9 (14.4)	98.8 (16.9)	95.2 (12.9)	84.4 (11.4)	< 0.0001
Low density lipoprotein (mg/dL)	128 (32)	128 (30)	125 (38)	125 (32)	128 (36)	0.78
High density lipoprotein (mg/dL)	54 (16)	55 (17)	56 (17)	53 (15)	53 (13)	0.61
Triglycerides (mg/dL)^†^	116 (86)	118 (84)	97 (64)	138 (113)	121 (89)	0.12
ADMA^‡ ^(μmol/L)	0.63 (0.10)	0.64 (0.11)	0.61 (0.10)	0.64 (0.09)	0.62 (0.09)	0.20
C-reactive protein (mg/L)	3.4 (6.4)	3.4 (6.7)	4.4 (6.6)	3.5 (5.1)	1.9 (2.8)	0.22
LpPLA_2 _mass (ng/mL)^†^	227 (71)	233 (75)	198 (63)	221 (59)	202 (73)	< 0.0001
LpPLA_2 _activity (nmol/ml-min)^†^	130 (43)	134 (41)	108 (32)	124 (36)	124 (61)	< 0.0001
LpPLA_2 _mass > 235 ng/mL	302 (42%)	260 (48%)	11 (18%)	19 (31%)	12 (24%)	< 0.0001
Current cigarette smoking	52 (7.3%)	42 (7.8%)	8 (13.3%)	1 (1.6%)	1 (1.9%)	0.035
Physical activity (kCal/kg-day)	34.8 (3.5)	34.9 (3.6)	34.4 (3.7)	35.0 (3.1)	34.6 (2.8)	0.76
Percent of calories from saturated fat	10.1 (2.7)	10.3 (2.7)	10.3 (2.9)	10.1 (2.5)	8.5 (1.7)	0.0001
Percent of calories from carbohydrates	46.6 (9.1)	46.0 (9.0)	46.3 (9.1)	49.1 (9.7)	49.8 (8.2)	0.0038
Annual income ≥ $50,000	452 (68%)	351 (70%)	37 (70%)	30 (54%)	34 (68%)	0.11
College graduate	173 (17%)	133 (25%)	10 (17%)	9 (15%)	21 (40%)	0.0059
Ala379Val						
Homozygous major	440 (63%)	340 (64%)	29 (49%)	32 (52%)	39 (75%)	0.011
Heterozygous	235 (33%)	170 (32%)	28 (47%)	25 (41%)	12 (23%)	0.021
Homozygous minor	28 (4%)	21 (4%)	2 (3%)	4 (7%)	1 (2%)	0.64
Arg92His						
Homozygous major	394 (56%)	291 (55%)	39 (66%)	27 (44%)	37 (71%)	0.011
Heterozygous	256 (36%)	198 (37%)	17 (29%)	28 (46%)	13 (25%)	0.075
Homozygous minor	55 (8%)	44 (8%)	3 (5%)	6 (10%)	2 (4%)	0.52
Ile198Thr						
Homozygous major	617 (87%)	479 (90%)	36 (61%)	59 (97%)	43 (83%)	< 0.0001
Heterozygous	85 (12%)	52 (10%)	22 (37%)	2 (3%)	9 (17%)	< 0.0001
Homozygous minor	4 (0.6%)	3 (0.6%)	1 (2%)	0 (0%)	0 (0%)	0.58

* P-value indicates the comparison across racial groups by ANOVA

^† ^Median (interquartile range), ^‡ ^ADMA, asymmetric dimethylarginine

Some baseline characteristics differed significantly among the racial groups (Table 1). The study had insufficient power to compare the non-White racial groups to each other, so all statistical comparisons are to Whites. Whites were the least likely to be diabetic, while in comparison, African-Americans were the most likely to be diabetic. Asians had the lowest mean BMI and smallest mean waist circumference. Asians and Hispanics were significantly less likely to be actively smoking as compared with Whites. Asians had a lower dietary intake of saturated fat and higher intake of from carbohydrates (both as percent of total energy). LDL and HDL levels were relatively similar comparing White with non-White groups. There were also significant differences in the SNP frequencies between White and non-White races. Compared with African-Americans and Hispanics, Whites were more likely to have the Ala379Val SNP. Hispanics were the most likely to have the Arg92His SNP, while African-Americans were the most likely to have the Ile198Thr SNP compared to Whites.

Men had significantly higher Lp-PLA_2 _mass and activity levels than women, after adjusting for all covariates (p = 0.025). The mean Lp-PLA_2 _mass for men was 246 ng/ml compared with 223 ng/ml for women. The mean Lp-PLA_2 _activity for men was 142 nmol/ml-min compared with 114 nmol/ml-min for women. There was no evidence of an interaction between sex and racial variations in Lp-PLA_2 _mass or activity in any model, so analyses were conducted in the combined cohort of men and women. A secondary analysis, which included subjects taking dyslipidemia medications, showed the same overall findings and confirmed that excluding these patients did not significantly change the final results.

**Figure 1:**
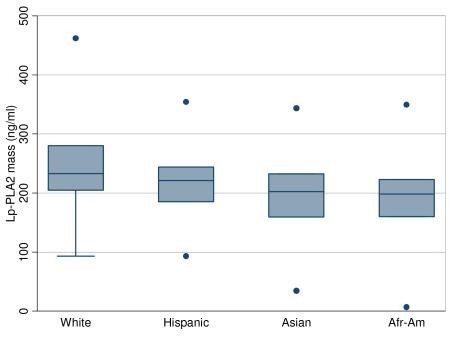
**Lp-PLA_2 _mass by race**. *Afr-Am*, African-American, The box plots ordered by median in descending order. The upper, middle, and lower line of the shaded box correspond to the 75th percentile, median, and 25th percentile values. The dots and the whiskers indicate minimum and maximum values. The dots are values that are greater than 1.5 times the interquartile range away from the 25th or 75th percentile.

**Figure 2:**
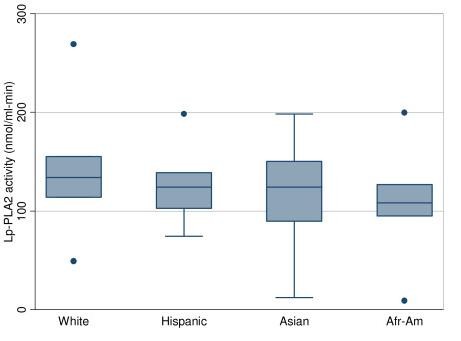
**Lp-PLA_2 _activity by race**. *Afr-Am*, African-American, The box plots ordered by median in descending order. The upper, middle, and lower line of the shaded box correspond to the 75th percentile, median, and 25th percentile values. The dots and the whiskers indicate minimum and maximum values. The dots are values that are greater than 1.5 times the interquartile range away from the 25th or 75th percentile.

Lp-PLA_2 _mass and activity levels varied significantly between Whites and non-Whites in all linear regression models (Figures 1 and 2 and Tables 2 and 3). Whites had the highest Lp-PLA_2 _mass and activity levels, followed by Hispanics. African-Americans and Asians had the lowest Lp-PLA_2 _mass and activity levels. These differences between Whites and non-Whites remained highly significant even after adjusting for all covariates. In fact, the values of the β coefficients for non-White races were high, ranging from -25 to -55 ng/ml across models for predicting Lp-PLA_2 _mass and -10 to -28 nmol/ml-min for predicting Lp-PLA_2 _activity. The other covariates with strong associations with Lp-PLA_2 _mass and activity levels included age, LDL, HDL, and ADMA levels. The Arg92His SNP in the PLA2G7 locus was associated with lower Lp-PLA_2 _activity, but higher Lp-PLA_2 _mass, whereas the other two SNPs had no significant associations.

**Table 2:** Linear regression models predicting Lp-PLA2 mass (ng/mL)

	Race & Sex (Model 1)		Model 1 + Biological Covariates		Model 1 + Lifestyle Covariates		Model 1 + SNPs*		All Covariates	
Variables	β	P-value	β	P-value	β	P-value	β	P-value	β	P-value
Race^†^										
African-American	-55.0	< 0.0001	-50.2	< 0.0001	-57.2	< 0.0001	-49.6	< 0.0001	-47.3	< 0.0001
Hispanic	-26.2	0.0016	-25.8	0.0008	-30.1	0.001	-27.7	0.0008	-30.0	0.0004
Asian	-47.1	< 0.0001	-45.7	< 0.0001	-45.4	< 0.0001	-43.1	< 0.0001	-42.0	< 0.0001
Female gender^‡^	-24.2	< 0.0001	-18.5	0.0045	-24.5	< 0.0001	-22.8	< 0.0001	-18.4	0.011
*Biological Covariates*										
Age (years)			2.4	0.0016					2.6	0.0019
Diabetes mellitus			-0.0042	0.95					-0.018	0.81
History of hypertension			-3.0	0.53					-3.76	0.49
Systolic blood pressure (mmHg)			0.16	0.23					0.14	0.33
Body mass index (kg/m2)			-0.46	0.63					-0.063	0.95
Waist circumference (cm)			0.092	0.81					-0.026	0.95
LDL (mg/dL)			0.75	< 0.0001					0.75	< 0.0001
HDL (mg/dL)			-0.26	0.094					-0.27	0.11
ADMA^§ ^(μmol/L)			38.8	0.06					33.2	0.14
C-reactive protein (mg/L)			1.1	0.0017					1.1	0.002
*Lifestyle/Demographic Covariates*										
Current smoking					0.012	0.90			0.0058	0.95
Exercise (kCal/kg-day)					0.63	0.40			0.76	0.28
% Calories from saturated fat					0.081	0.94			-0.96	0.38
% Calories from carbohydrates					-0.43	0.20			-0.60	0.058
Annual income ≥ $50,000					-0.019	0.74			0.051	0.32
College graduate					-0.091	0.086			-0.071	0.15
*SNPs*										
Ala379Val							2.2	0.60	2.5	0.55
Arg92His							11.8	0.0023	11.6	0.0025
Ile198Thr							-8.8	0.19	-6.1	0.37

* SNPs, single nucleotide polymorphisms

^† ^Compared to White race

^‡ ^Compared to Male

^§^ADMA, asymmetric dimethylarginine

**Table 3:** Linear regression models predicting Lp-PLA2 activity (nmol/mL-min)

	Race & Sex (Model1)		Model1 + Biological Covariates		Model 1 + Lifestyle Covariates		Model1 + SNPs*		All Covariates	
Variables	β	P-value	β	P-value	β	P-value	β	P-value	β	P-value
Race^†^										
African-American	-24.7	< 0.0001	-18.5	< 0.0001	-27.0	< 0.0001	-22.8	< 0.0001	-18.0	< 0.0001
Hispanic	-10.1	0.009	-10.6	0.0006	-14.4	0.0006	-10.2	0.009	-13.1	0.0001
Asian	-12.1	0.003	-14.2	< 0.0001	-10.4	0.018	-11.9	0.004	-13.9	0.0001
Female gender^‡^	-28.1	< 0.0001	-18.2	< 0.0001	-28.3	< 0.0001	-27.6	< 0.0001	-17.4	< 0.0001
*Biological Covariates*										
Age (years)			1.05	0.0006					1.13	0.0006
Diabetes mellitus			-0.057	0.038					-0.067	0.024
History of hypertension			-2.38	0.23					-3.65	0.094
Systolic blood pressure (mmHg)			-0.0034	0.95					0.0012	0.98
Body mass index (kg/m2)			-0.41	0.29					-0.34	0.39
Waist circumference (cm)			0.041	0.79					-0.0066	0.97
LDL (mg/dL)			0.44	< 0.0001					0.45	< 0.0001
HDL (mg/dL)			-0.69	< 0.0001					-0.72	< 0.0001
ADMA^§ ^(μmol/L)			23.4	0.0048					23.1	0.012
C-reactive protein (mg/L)			-0.23	0.10					-0.22	0.13
*Lifestyle/Demographic Covariates*										
Current smoking					0.035	0.43			0.025	0.49
Exercise (kCal/kg-day)					0.36	0.30			0.38	0.17
% Calories from saturated fat					1.09	0.038			0.047	0.91
% Calories from carbohydrates					0.31	0.045			-0.055	0.66
Annual income ≥ $50,000					-0.023	0.38			0.011	0.60
College graduate					-0.0095	0.70			0.014	0.48
*SNPs*										
Ala379Val							1.20	0.55	1.45	0.40
Arg92His							-2.06	0.26	-3.75	0.015
Ile198Thr							-6.03	0.056	-4.38	0.11

* SNPs, single nucleotide polymorphisms

^† ^Compared to White race

^‡ ^Compared to Male

^§^ADMA, asymmetric dimethylarginine

None of the other covariates were consistently associated with Lp-PLA_2 _mass and activity levels. Hypertension and diabetes were weakly associated with Lp-PLA_2 _activity and not associated with mass. Conversely, C-reactive protein was weakly associated with Lp-PLA_2 _mass, but not activity. None of the lifestyle covariates were significantly associated with Lp-PLA_2 _mass or activity levels.

## Discussion

Our study confirms that Lp-PLA_2 _mass and activity levels vary significantly between White and non-White races in healthy older adults without clinically diagnosed CHD and not taking dyslipidemia medications. Whites have the highest Lp-PLA_2 _mass and activity levels, Hispanics have intermediate levels, and African-Americans and Asians have the lowest Lp-PLA_2 _mass and activity levels. These relationships were not affected by statistical adjustment for lifestyle and demographic factors, standard biological risk factors, and three SNPs known to be associated with Lp-PLA_2 _activity levels. These findings suggest that differences in Lp -PLA_2 _mass and activity levels between Whites and non-Whites may be due to other genetic factors or perhaps to unmeasured lifestyle factors.

The differences between White, Hispanic, and African-American race and Lp-PLA2 mass and activity levels in our study were similar to the results from the Dallas Heart Study, the largest community-based study of Lp-PLA_2 _levels[11]. One key difference was that our study also included Asians, who were found to have Lp-PLA_2 _mass and activity levels that were similar to African-Americans, but lower than Whites. Our study also demonstrated that racial differences in Lp-PLA_2 _mass and activity levels apply to older adults, given the fact that individuals in our study (mean age of 66 years) were substantially older than those in the Dallas Heart Study (mean age of 45 years). The Dallas Heart Study cohort included individuals taking statin medication, whereas our study excluded individuals taking any dyslipidemia medication, because such medications may reduce Lp-PLA_2 _mass and activity levels[25]. Other studies that were in a small cohort[26] and in a selected population with vascular disease[27] also showed that Whites have higher Lp-PLA_2 _mass and activity levels as compared with other races.

Because Lp-PLA_2 _circulates bound to LDL particles, Lp-PLA_2 _mass and activity levels depend greatly upon lipid metabolism and are strongly association with LDL and HDL levels[16]. Although we also found this association, adjustment for LDL and HDL levels did not alter the relation between race and Lp-PLA_2 _mass and activity levels, suggesting that variations in LDL and HDL levels do not explain these racial differences. Diet and physical activity also affect lipid metabolism,[28] but adjustment for these lifestyle factors did not attenuate the association between race and Lp-PLA_2 _mass and activity levels.

Various studies have shown associations between Lp-PLA_2 _mass and activity levels and different genotypes of Lp-PLA_2_, bolstering the hypothesis that there is a substantial genetic influence on Lp-PLA_2 _levels. A study of the Framingham cohort, using 1943 SNPs, suggested that Lp-PLA_2 _mass had a multivariable adjusted heritability of 25% and Lp-PLA_2 _activity had 41% heritability[29]. Among Whites, the Ala379Val coding variant in the PLA2G7 locus was associated with increased Lp-PLA_2 _activity, while the Arg92His coding variant was associated with decreased activity[22]. Among Japanese individuals, the Val279Phe SNP also in the PLA2G7 locus, which is much more prevalent in Japanese than in Whites, is associated with greatly decreased Lp-PLA_2 _activity[30]. In fact, people who are homozygous for the Val279Phe polymorphism have almost no detectable Lp-PLA_2 _activity[31,32]. Because of the probable strong genetic influence on Lp-PLA_2 _mass and activity levels, one could hypothesize that the racial variations are due to genetic differences between races. Our study included only three SNPs, while the Framingham study revealed 12 SNPs linked with Lp-PLA_2 _variability[29]. Therefore, adjusting for only three SNPs in our study was a limited test of genetic influence on the racial differences in Lp-PLA_2 _and did not attenuate the strong association between race and Lp-PLA_2 _mass and activity levels.

In addition to the limited number of Lp-PLA_2_-related SNPs, another limitation of this analysis is that it is cross-sectional as we were unable to study whether Lp-PLA_2 _level predicts subsequent risk of CHD events differentially by race. Before Lp-PLA_2 _results can be added to existing CHD risk prediction equations, further evidence is needed to validate its incremental prognostic value in an ethnically-diverse cohort. Another limitation is the ability of questionnaires to accurately and fully characterize all the lifestyle variations that could explain the racial differences of Lp-PLA_2 _mass and activity levels. For example, there could be other lifestyle factors, such as diet choices unrelated to fat and carbohydrate intake, which influence Lp-PLA_2 _mass and activity levels but are not captured by the survey. The relatively modest number of non-White participants may also limit our ability to detect the effects of lifestyle factors.

Lp-PLA_2 _is viewed as a novel CHD risk marker that could be used to risk stratify individuals when considering primary prevention strategies, such as initiating statin therapy or setting lipid treatment goals. Various authors have suggested using a uniform threshold for Lp-PLA_2 _mass, regardless of sex and race, for categorizing risk[1,33]. However, the results from this study confirm that Lp-PLA_2 _mass and activity levels vary significantly between Whites and non-Whites, independent of known biological and lifestyle factors. These racial variations could affect the performance of Lp-PLA_2 _as a prognostic risk marker. For example, African-Americans may have lower Lp-PLA_2 _mass and activity levels than Whites despite having similar degrees of atherosclerosis and vascular inflammation or they may simply have less underlying atherosclerotic disease. In light of the high cardiovascular event rates in African-Americans, the former possibility seems more likely. As a result, using the same Lp-PLA_2 _threshold in Whites and African-Americans may underestimate risk in African-Americans compared with Whites.

## Conclusions

If Lp-PLA_2 _mass and activity levels are inherently different between races, then these levels may need to be interpreted differently for various races. Before incorporating information on Lp-PLA_2 _mass and activity levels in assessing cardiovascular risk for individual patients, additional larger, multi-racial prospective studies are needed to delineate the specific risk associated with different Lp-PLA_2 _levels within individual racial groups.

## Competing interests

The authors declare that they have no competing interests.

## Authors' contributions

SPF, JMF, CI, ASG, MAH, and TQ conceived of the study. AV, KKL, JMF, SPF, and CI had input to the statistical analyses. KKL drafted the manuscript. All authors edited the manuscript, approved the analyses, and read and approved the final manuscript.

## Pre-publication history

The pre-publication history for this paper can be accessed here:

http://www.biomedcentral.com/1471-2261/11/38/prepub

## References

[B1] Corson MA Jones PH Davidson MH Review of the evidence for the clinical utility of lipoprotein-associated phospholipase A2 as a cardiovascular risk marker Am J Cardiol 2008 101 41F 50F 10.1016/j.amjcard.2008.04.018 18549871

[B2] Garza CA Montori VM McConnell JP Somers VK Kullo IJ Lopez-Jimenez F Association between lipoprotein-associated phospholipase A2 and cardiovascular disease: a systematic review Mayo Clin Proc 2007 82 159 65 10.4065/82.2.159 17290721

[B3] Ballantyne CM Hoogeveen RC Bang H Coresh J Folsom AR Heiss G Sharrett AR Lipoprotein-associated phospholipase A2, high-sensitivity C-reactive protein, and risk for incident coronary heart disease in middle-aged men and women in the Atherosclerosis Risk in Communities (ARIC) study Circulation 2004 109 837 42 10.1161/01.CIR.0000116763.91992.F1 14757686

[B4] Koenig W Khuseyinova N Lowel H Trischler G Meisinger C Lipoprotein-associated phospholipase A2 adds to risk prediction of incident coronary events by C-reactive protein in apparently healthy middle-aged men from the general population: results from the 14-year follow-up of a large cohort from southern Germany Circulation 2004 110 1903 8 10.1161/01.CIR.0000143377.53389.C8 15451783

[B5] Oei HH van der Meer IM Hofman A Koudstaal PJ Stijnen T Breteler MM Witteman JC Lipoprotein-associated phospholipase A2 activity is associated with risk of coronary heart disease and ischemic stroke: the Rotterdam Study Circulation 2005 111 570 5 10.1161/01.CIR.0000154553.12214.CD 15699277

[B6] Packard CJ O'Reilly DS Caslake MJ McMahon AD Ford I Cooney J Macphee CH Suckling KE Krishna M Wilkinson FE Rumley A Lowe GD Lipoprotein-associated phospholipase A2 as an independent predictor of coronary heart disease. West of Scotland Coronary Prevention Study Group N Engl J Med 2000 343 1148 55 10.1056/NEJM200010193431603 11036120

[B7] Ballantyne CM Hoogeveen RC Bang H Coresh J Folsom AR Chambless LE Myerson M Wu KK Sharrett AR Boerwinkle E Lipoprotein-associated phospholipase A2, high-sensitivity C-reactive protein, and risk for incident ischemic stroke in middle-aged men and women in the Atherosclerosis Risk in Communities (ARIC) study Arch Intern Med 2005 165 2479 84 10.1001/archinte.165.21.2479 16314544

[B8] Iribarren C Lipoprotein-Associated Phospholipase A2 and C-Reactive Protein for Measurement of Inflammatory Risk: Independent or Complementary? Cardiovascular Risk Reports 2010

[B9] Thompson A Gao P Orfei L Watson S Di Angelantonio E Kaptoge S Ballantyne C Cannon CP Criqui M Cushman M Hofman A Packard C Thompson SG Collins R Danesh J Lipoprotein-associated phospholipase A(2) and risk of coronary disease, stroke, and mortality: collaborative analysis of 32 prospective studies Lancet 2010 375 1536 44 20435228 10.1016/S0140-6736(10)60319-4 PMC2864403

[B10] White H Held C Stewart R Watson D Harrington R Budaj A Steg PG Cannon CP Krug-Gourley S Wittes J Trivedi T Tarka E Wallentin L Study design and rationale for the clinical outcomes of the STABILITY Trial (STabilization of Atherosclerotic plaque By Initiation of darapLadIb TherapY) comparing darapladib versus placebo in patients with coronary heart disease Am Heart J 2010 160 655 61 10.1016/j.ahj.2010.07.006 20934559

[B11] Brilakis ES Khera A McGuire DK See R Banerjee S Murphy SA de Lemos JA Influence of race and sex on lipoprotein-associated phospholipase A2 levels: observations from the Dallas Heart Study Atherosclerosis 2008 199 110 5 10.1016/j.atherosclerosis.2007.10.010 18061193 PMC2495009

[B12] Fair JM Kiazand A Varady A Mahbouba M Norton L Rubin GD Iribarren C Go AS Hlatky MA Fortmann SP Ethnic differences in coronary artery calcium in a healthy cohort aged 60 to 69 years Am J Cardiol 2007 100 981 5 10.1016/j.amjcard.2007.04.038 17826382

[B13] Taylor-Piliae RE Norton LC Haskell WL Mahbouda MH Fair JM Iribarren C Hlatky MA Go AS Fortmann SP Validation of a new brief physical activity survey among men and women aged 60-69 years Am J Epidemiol 2006 164 598 606 10.1093/aje/kwj248 16840522

[B14] Assimes TL Knowles JW Priest JR Basu A Borchert A Volcik KA Grove ML Tabor HK Southwick A Tabibiazar R Sidney S Boerwinkle E Go AS Iribarren C Hlatky MA Fortmann SP Myers RM Kuhn H Risch N Quertermous T A near null variant of 12/15-LOX encoded by a novel SNP in ALOX15 and the risk of coronary artery disease Atherosclerosis 2008 198 136 44 10.1016/j.atherosclerosis.2007.09.003 17959182 PMC2440699

[B15] Sydow K Fortmann SP Fair JM Varady A Hlatky MA Go AS Iribarren C Tsao PS Distribution of asymmetric dimethylarginine among 980 healthy, older adults of different ethnicities Clin Chem 2009 56 111 20 19892843 10.1373/clinchem.2009.136200

[B16] Brilakis ES McConnell JP Lennon RJ Elesber AA Meyer JG Berger PB Association of lipoprotein-associated phospholipase A2 levels with coronary artery disease risk factors, angiographic coronary artery disease, and major adverse events at follow-up Eur Heart J 2005 26 137 44 15618069 10.1093/eurheartj/ehi010

[B17] Koenig W Twardella D Brenner H Rothenbacher D Lipoprotein-associated phospholipase A2 predicts future cardiovascular events in patients with coronary heart disease independently of traditional risk factors, markers of inflammation, renal function, and hemodynamic stress Arterioscler Thromb Vasc Biol 2006 26 1586 93 10.1161/01.ATV.0000222983.73369.c8 16627803

[B18] Blair SN Haskell WL P Ho Paffenbarger RS Vranizan KM Farquhar JW Wood PD Assessment of habitual physical activity by a seven-day recall in a community survey and controlled experiments Am J Epidemiol 1985 122 794 804 3876763 10.1093/oxfordjournals.aje.a114163

[B19] Sallis JF Haskell WL Wood PD Fortmann SP Rogers T Blair SN Paffenbarger RS Jr Physical activity assessment methodology in the Five-City Project Am J Epidemiol 1985 121 91 106 3964995 10.1093/oxfordjournals.aje.a113987

[B20] Block G Hartman AM Dresser CM Carroll MD Gannon J Gardner L A data-based approach to diet questionnaire design and testing Am J Epidemiol 1986 124 453 69 3740045 10.1093/oxfordjournals.aje.a114416

[B21] Zakharia F Basu A Absher D Assimes TL Go AS Hlatky MA Iribarren C Knowles JW J Li Narasimhan B Sidney S Southwick A Meyers RM Quertermous T Risch N Tang H Characterizing the admixed African ancestry of African Americans Genome Biol 2009 10 R141 10.1186/gb-2009-10-12-r141 20025784 PMC2812948

[B22] Hoffmann MM Winkler K Renner W Winkelmann BR Seelhorst U Wellnitz B Boehm BO Marz W Genetic variants and haplotypes of lipoprotein associated phospholipase A2 and their influence on cardiovascular disease (The Ludwigshafen Risk and Cardiovascular Health Study) J Thromb Haemost 2009 7 41 8 10.1111/j.1538-7836.2008.03216.x 18983494

[B23] Cowie CC Rust KF Ford ES Eberhardt MS Byrd-Holt DD C Li Williams DE Gregg EW Bainbridge KE Saydah SH Geiss LS Full accounting of diabetes and pre-diabetes in the U.S. population in 1988-1994 and 2005-2006 Diabetes Care 2009 32 287 94 19017771 10.2337/dc08-1296 PMC2628695

[B24] Wolf-Maier K Cooper RS Banegas JR Giampaoli S Hense HW Joffres M Kastarinen M Poulter N Primatesta P Rodriguez-Artalejo F Stegmayr B Thamm M Tuomilehto J Vanuzzo D Vescio F Hypertension prevalence and blood pressure levels in 6 European countries, Canada, and the United States JAMA 2003 289 2363 9 10.1001/jama.289.18.2363 12746359

[B25] Albert MA Glynn RJ Wolfert RL Ridker PM The effect of statin therapy on lipoprotein associated phospholipase A2 levels Atherosclerosis 2005 182 193 8 10.1016/j.atherosclerosis.2005.05.006 15982658

[B26] El-Saed A Sekikawa A Zaky RW Kadowaki T Takamiya T Okamura T Edmundowicz D Kita Y Kuller LH Ueshima H Association of lipoprotein-associated phospholipase A2 with coronary calcification among American and Japanese men J Epidemiol 2007 17 179 85 10.2188/jea.17.179 18094516 PMC3659786

[B27] Allison MA Denenberg JO Nelson JJ Natarajan L Criqui MH The association between lipoprotein-associated phospholipase A2 and cardiovascular disease and total mortality in vascular medicine patients J Vasc Surg 2007 46 500 6 10.1016/j.jvs.2007.04.038 17681710 PMC2700305

[B28] Stefanick ML Mackey S Sheehan M Ellsworth N Haskell WL Wood PD Effects of diet and exercise in men and postmenopausal women with low levels of HDL cholesterol and high levels of LDL cholesterol N Engl J Med 1998 339 12 20 10.1056/NEJM199807023390103 9647874

[B29] Schnabel R Dupuis J Larson MG Lunetta KL Robins SJ Zhu Y Rong J Yin X Stirnadel HA Nelson JJ Wilson PW Keaney JF Vasan RS Benjamin EJ Clinical and genetic factors associated with lipoprotein-associated phospholipase A2 in the Framingham Heart Study Atherosclerosis 2009 204 601 7 10.1016/j.atherosclerosis.2008.10.030 19135199 PMC2893025

[B30] Zhang SY Shibata H Karino K Wang BY Kobayashi S Masuda J Nabika T Comprehensive evaluation of genetic and environmental factors influencing the plasma lipoprotein-associated phospholipase A2 activity in a Japanese population Hypertens Res 2007 30 403 9 10.1291/hypres.30.403 17587752

[B31] Wang T Karino K Yamasaki M Zhang Y Masuda J Yamaguchi S Shiwaku K Nabika T Effects of G994T in the Lp-PLA2 gene on the plasma oxidized LDL level and carotid intima-media thickness in Japanese: the Shimane study Am J Hypertens 2009 22 742 7 10.1038/ajh.2009.70 19373214

[B32] Yamada Y Yoshida H Ichihara S Imaizumi T Satoh K Yokota M Correlations between plasma platelet-activating factor acetylhydrolase (PAF-AH) activity and PAF-AH genotype, age, and atherosclerosis in a Japanese population Atherosclerosis 2000 150 209 16 10.1016/S0021-9150(99)00385-8 10781653

[B33] Lanman RB Wolfert RL Fleming JK Jaffe AS Roberts WL Warnick GR McConnell JP Lipoprotein-associated phospholipase A2: review and recommendation of a clinical cut point for adults Prev Cardiol 2006 9 138 43 10.1111/j.1520-037X.2006.05547.x 16849876

